# Effect of hypertension on outcomes of adult inpatients with COVID-19 in Wuhan, China: a propensity score–matching analysis

**DOI:** 10.1186/s12931-020-01435-8

**Published:** 2020-07-06

**Authors:** Qing Yang, Ying Zhou, Xinrong Wang, Shan Gao, Yang Xiao, Weiming Zhang, Yi Hu, Yafei Wang

**Affiliations:** Department of Respiratory and Critical Care Medicine, The Central Hospital of Wuhan, Tongji Medical College, Huazhong University of Science and Technology, No. 26 Shengli Street, Wuhan, 430014 China

**Keywords:** COVID-19, Hypertension, Mortality, Propensity score–matching analysis

## Abstract

**Background:**

Previous studies have shown that Coronavirus Disease 2019 (COVID-19) patients with underlying comorbidities can have worse outcomes. However, the effect of hypertension on outcomes of COVID-19 patients remains unclear.

**Research question:**

The aim of this study was to explore the effect of hypertension on the outcomes of patients with COVID-19 by using propensity score–matching (PSM) analysis.

**Study design and methods:**

Participants enrolled in this study were patients with COVID-19 who had been hospitalized at the Central Hospital of Wuhan, China. Chronic comorbidities and laboratory and radiological data were reviewed; patient outcomes and lengths of stay were obtained from discharge records. We used the Cox proportional-hazard model (CPHM) to analyze the effect of hypertension on these patients’ outcomes and PSM analysis to further validate the abovementioned effect.

**Results:**

A total of 226 patients with COVID-19 were enrolled in this study, of whom 176 survived and 50 died. The proportion of patients with hypertension among non-survivors was higher than that among survivors (26.70% vs. 74.00%; *P* < 0.001). Results obtained via CPHM showed that hypertension could increase risk of mortality in COVID-19 patients (hazard ratio 3.317; 95% CI [1.709–6.440]; *P* < 0.001). Increased D-dimer levels and higher ratio of neutrophils to lymphocytes (N/L) were also found to increase these patients’ mortality risk. After matching on propensity score, we still came to similar conclusions. After we applied the same method in critically ill patients, we found that hypertension also increased risk of death in patients with severe COVID-19.

**Conclusion:**

Hypertension, increased D-dimer and the ratio of neutrophil to lymphocyte increased mortality in patients with COVID-19, with hypertension in particular.

## Introduction

In December 2019, a pneumonia caused by an unknown pathogen broke out in Wuhan City, the capital of Hubei Province, China. In January 2020, severe acute respiratory syndrome coronavirus 2 (SARS-CoV-2) was identified as the pathogen underlying the disease. SARS-CoV-2 forms a unique clade of the *Sarbecovirus* subgroup of the *Orthocoronavirus* subfamily, having emerged as the seventh coronavirus that can infect human beings [1] . As of March 23, 2020, the number of confirmed cases of coronavirus disease 2019 (COVID-19) caused by SARS-CoV-2 has surpassed 300,000 globally, resulting in more than 14,000 deaths [2] .

Previous studies have shown that patients with underlying comorbidities can have worse outcomes, and some laboratory indicators are also associated with mortality [3, 4] . Some studies have shown that hypertension is a risk factor for patients with severe COVID-19 [5] . There have been more patients with hypertension among those who have died than among survivors. However, the effect of hypertension on outcomes of COVID-19 patients is not completely clear or uniform. Therefore, we designed this study to investigate the effect of hypertension on the outcomes of these patients, using the propensity score–matching (PSM) method to eliminate the influence of other confounding factors.

## Methods

### Study design and participants

This single-center, retrospective cohort study was conducted at the Central Hospital of Wuhan, a tertiary teaching hospital that is responsible for the treatment for patients of COVID-19 as assigned by the Chinese government. All participants included in the study were local residents of Wuhan who were hospitalized at Central Hospital of Wuhan from January 1 to February 29, 2020. Relevant laboratory examinations, common-pathogen detection, and chest computed-tomography (CT) scanning were conducted. Each patient also underwent the SARS-CoV-2 nucleic-acid swab test, with some patients testing positive and others negative. The immaturity of the methods and an incorrect sampling method used for SARS-CoV-2 nucleic-acid detection might have contributed to false-negative results. High-resolution CT scans with a scan layer thickness of 5 mm and reconstruction of a 1- to 1.5-mm thick layer are recommended for radiological examination of COVID-19 patients. The included patients were clinically diagnosed with COVID-19 according to the World Health Organization (WHO)‘s interim guidelines [6] . For patients who were suspected to have the illness, two senior respiratory doctors made a joint diagnosis.

We divided the participants in our study into two groups: survivors and non-survivors. Critically ill patients were defined as those who had the following clinical signs: fever or suspected respiratory infection plus one or more of the following: respiratory rate > 30 breaths/min, severe respiratory distress, or oxygen saturation (SpO2) < 90% on room air. Patients with acute respiratory distress syndrome (ARDS), sepsis, or septic shock were also included. Patients with other viral, bacterial, or fungal infections on admission and those with missing data were excluded. Participants with hypertension in the study had been previously diagnosed with same by a clinician and regularly took oral antihypertensive medications over a long period of time. The outcomes observed in the study were either in-hospital death or discharge from the hospital.

This study was approved by the Ethics Committee of Wuhan Central Hospital. As it was a retrospective study and did not require protection of patients’ privacy, we were exempted from obtaining informed consent.

### Data collection

We reviewed electronic medical records, from which we obtained chronic comorbidities, demographic data, laboratory examination results, and chest CT images. Data on counts or levels of white blood cells (WBCs), lymphocytes (L), neutrophils (N), alanine transaminase (ALT), aspartate aminotransferase (AST), serum albumin (ALB), serum creatinine (sCr), blood urea nitrogen (BUN), D-dimer, and C-reactive protein (CRP) were included. Diagnoses of hypertension came from the electronic medical records system. Length of hospital stay was obtained from discharge records, from which we also learned the patient’s outcome. Scores on the Acute Physiology and Chronic Health Evaluation II (APACHE II) had been calculated for critically ill patients within 24 h of admission. We obtained dosage and duration of hormone use from physician medication order entry systems; the physicians had acquired these data via an examination on the first day after admission. All data were checked by another researcher to ascertain their accuracy.

### Statistical analysis

Continuous variables were presented as means (standard deviation [SD]) and compared using *t* tests if they were normally distributed, or described using medians (interquartile ranges [IQRs]) if they were not.. The Mann–Whitney *U* test was used for comparisons. We expressed descriptive data as number (%) for categorical variables and compared them via chi-square test or Fisher’s exact test. We used the Kaplan–Meier (K–M) method to evaluate the relationships between variables and outcome and Cox proportional-hazard model (CPHM) analysis to clarify the effects of each factor on outcome. A two-sided α value of < 0.05 was considered to be statistically significant. We analyzed all data using SSPS software version 22.0 (IBM Corp., Armonk, New York, US).

### Propensity score matching

After performing statistical analysis as described above, we conducted PSM analysis to minimize the effect of selection bias and to control for potential confounding factors. PSM analysis attempts to compare outcomes between patients who have similar distributions of all covariates measured, thus enabling further clarification of variables’ effects on outcome. Matching was performed using a 1:1 matching protocol without replacement (greedy-matching algorithm), with caliper width = 0.2 of the standard deviation of the logit of the propensity score. We further adjusted for the comparative risks of outcomes in the matched cohort using a CPHM.

## Results

### Baseline characteristics

A total of 226 patients with COVID-19 were enrolled in our analysis from January 1 to February 29, 2020. Of these, 111 (49.12%) tested positive for SARS-CoV-2 nucleic acids. Non-survivors accounted for 22.12% of all participants. The basic characteristics of the study population are shown in Table 1. Non-survivors tended to be older and to have chronic comorbidities such as hypertension, type 2 diabetes mellitus (T2DM), and coronary heart disease (CHD). However, there was no significant difference in the proportion of patients with chronic obstructive pulmonary disease (COPD) between the two groups. Compared with survivors, non-survivors had significantly higher serum N counts and higher levels of CRP, AST, sCr, BUN, and D-dimer. Serum ALB levels and L counts of non-survivors were lower than those of survivors. The ratio of neutrophils to lymphocytes (N/L) was significantly higher in non-survivors. In addition, no significant differences in sex, ALT levels, or time from onset to admission were apparent.

**Table 1:** Baseline characteristics of all patients with COVID-19

	Survivor(*n* = 176)	Non-survivor(*n* = 50)	*P*-value
Males (%)	82 (46.60%)	31 (62%)	0.077
Age (years)	49.93 (15.35)	67.82 (15.73)	< 0.001
Onset time (day)	7.16 (3.86)	6.54 (3.71)	0.619
Hypertension (%)	47 (26.70%)	37 (74%)	< 0.001
T2DM (%)	28 (15.91%)	17 (34%)	0.008
CHD (%)	6 (3.41%)	7 (14%)	0.010
COPD (%)	3 (1.70%)	3 (6.00%)	0.124
WBC (10^9^)	5.15 (3.71–6.44)	5.73 (4.18–7.95)	0.078
N (10^9^)	3.34 (2.34–4.76)	4.32 (2.97–7.05)	0.002
L (10^9^)	1.10 (0.75–1.52)	0.66 (0.46–0.92)	< 0.001
N/L	2.98 (1.70–5.51)	6.18 (3.58–12.78)	< 0.001
ALT (U/L)	19.70 (13.60–33.65)	21.05 (14.33–29.48)	0.819
AST (U/L)	21.35 (17.20–32.95)	31.50 (22.40–44.33)	0.001
ALB (g/L)	39.24 (5.25)	35.55 (4.30)	< 0.001
sCr (μmol/L)	64.90 (52.20–75.93)	76.00 (58.00–109.20)	0.003
BUN (mmol/L)	3.97 (3.13–5.25)	6.60 (4.60–10.58)	< 0.001
CRP (mg/dL)	1.52 (0.28–5.03)	6.10 (3.53–8.84)	< 0.001
D-dimer (μg/mL)	0.56 (0.28–1.28)	6.21 (1.27–19.73)	< 0.001

Abbreviations: Onset time, time from onset to admission (days); *T2DM* Type 2 diabetes mellitus; *CHD* Coronary heart disease; *COPD* Chronic obstructive pulmonary disease; *WBC* White blood cells; *L* Lymphocyte count; *N* Neutrophil count; *N/L* Ratio of neutrophils to lymphocytes; *ALT* alanine transaminase; *AST* Aspartate aminotransferase; *ALB* Serum albumin; *sCr* Serum creatinine; *BUN* Blood urea nitrogen; *CRP* C-reactive protein

### Univariate Kaplan–Meier analysis

Before the multivariate Cox regression analysis, we performed K–M analysis to screen out the appropriate variables in order to make the results more accurate. Categorical variables with statistically significant differences between the two groups were directly analyzed by K–M analysis, and continuous variables were converted into categorical variables according to the median number before K–M analysis. After K–M analysis, we found that age, hypertension, T2DM, N/L, CRP, AST, ALB, BUN and D-dimer had effects on patient outcomes (Table S1 in the Supplementary Appendix).

### Cox-proportional hazard model analysis

**Fig. 1:**
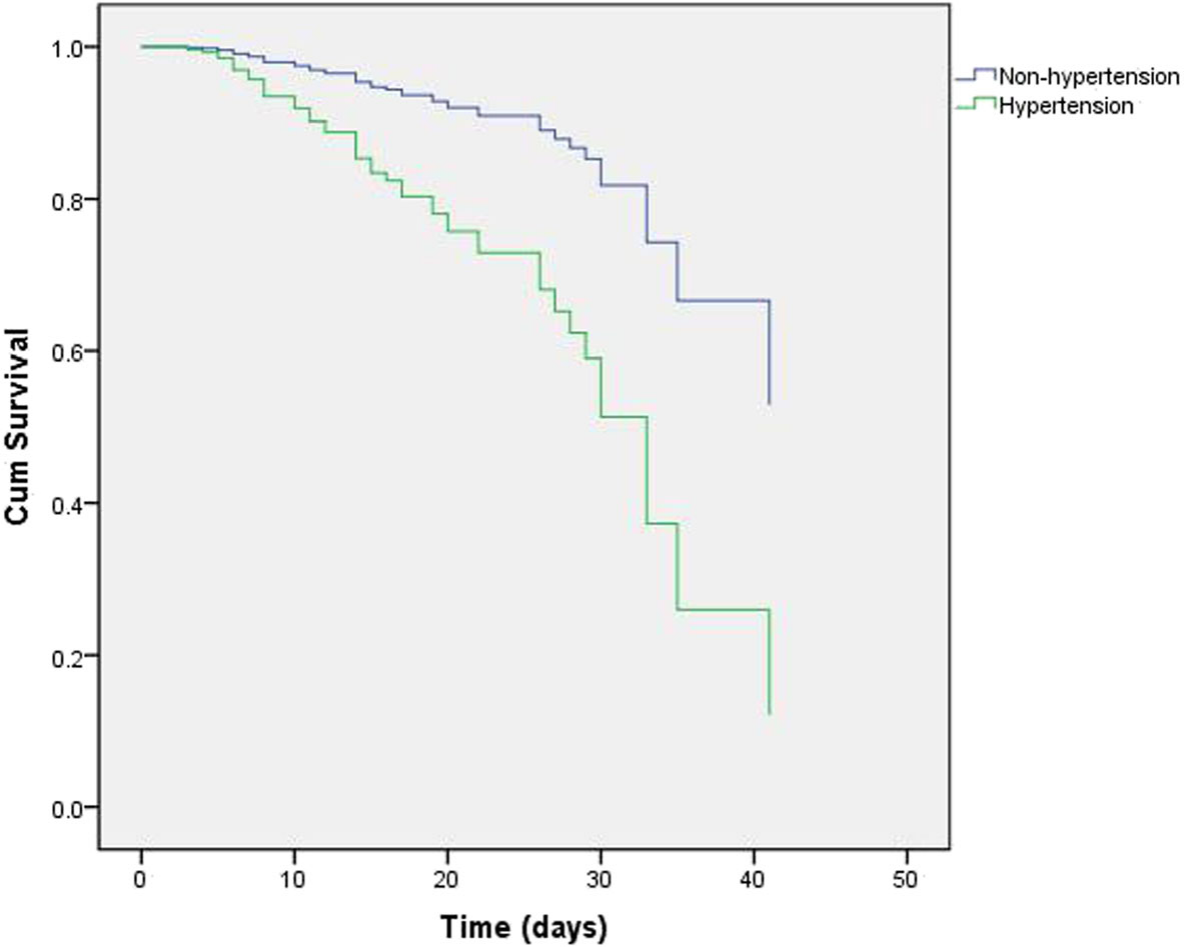
Cox regression survival plot according to hypertension for patients with COVID-19. Hypertension: hazard ratio (HR), 3.317; 95% confidence interval (CI), 1.709–6.440; *P* < 0.001

We conducted multivariate CPHM analysis to further certify whether variables affected patient outcomes. Those that did so were incorporated into CPHM analysis, as were sex and sCr. The results demonstrated that patients with hypertension had a higher mortality risk (hazard ratio [HR] 3.317; 95% CI [1.709–6.440]; *P* < 0.001; Fig. 1). In addition, elevated D-dimer levels and higher N/L might also have increased patients’ risk of death in (Table 2).

**Table 2:** Results of Cox proportional-hazard model analysis

	HR	95% CI	*P*-value
Hypertension	3.317	(1.709–6.440)	< 0.001
D-dimer	1.026	(1.016–1.036)	< 0.001
N/L	1.103	(1.060–1.148)	< 0.001

Abbreviations: *HR* Hazard ratio; *CI* Confidence interval; *N/L*, ratio of neutrophils to lymphocytes. The following variables were incorporated into Cox proportional-hazard model analysis: Serum creatinine (*sCr*), Blood urea nitrogen (*BUN*), Sex, type 2 diabetes mellitus (*T2DM*), Serum albumin (*ALB*), C-reactive protein (*CRP*), Age, aspartate aminotransferase (*AST*), Hypertension, D-dimer, and N/L.

### Results of analysis in critically ill patients

**Fig. 2:**
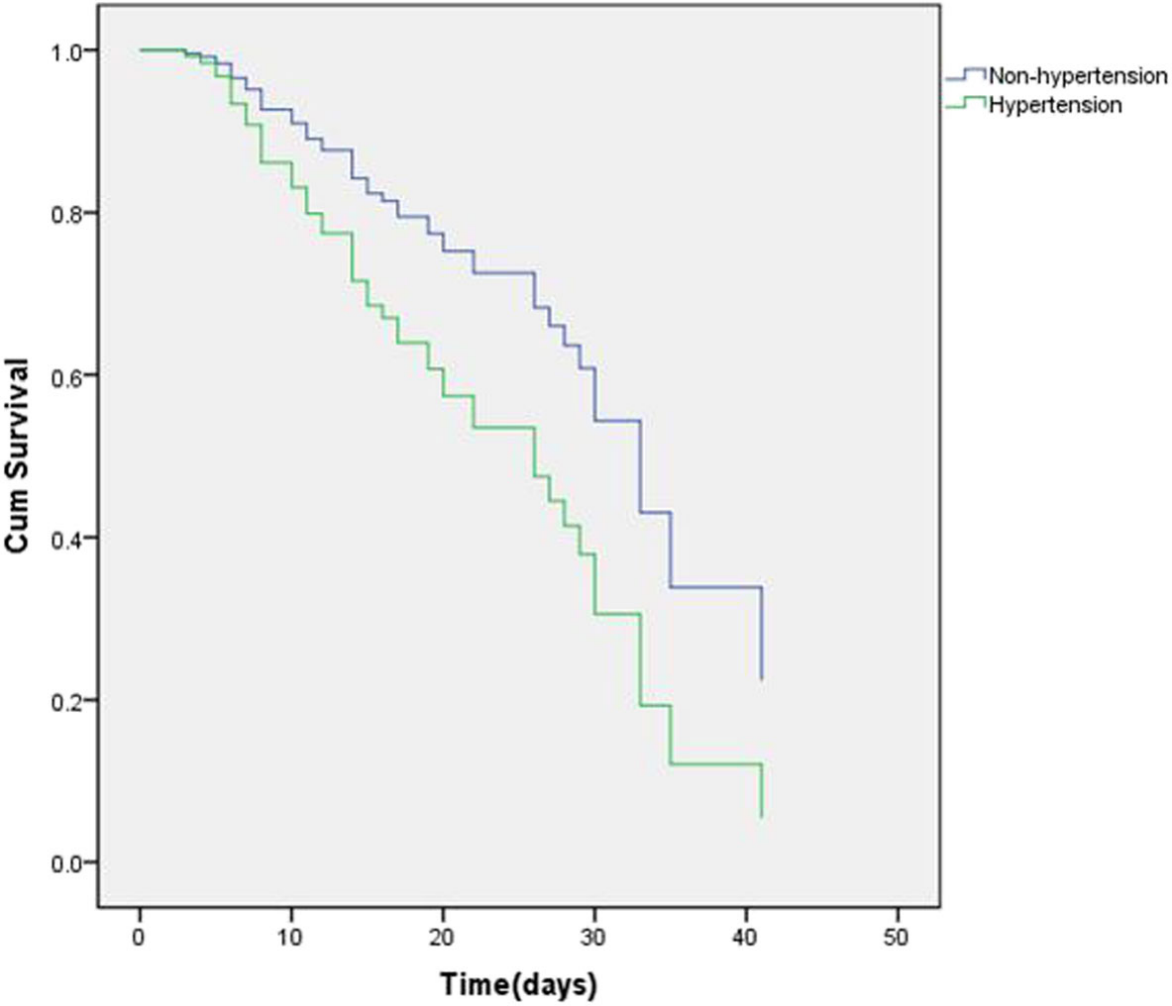
Cox regression survival plot according to hypertension for critically ill patients with COVID-19

The basic characteristics of critically ill patients was listed (Table S2 in the Supplementary Appendix). Of these patients, 47.62% died. Non-survivors were older, and more non-survivors than survivors had hypertension (45.45% vs. 74.00%; *P* = 0.005). N/L and D-dimer levels were significantly higher in non-survivors than in survivors. Hormone use was slightly different between the two groups. All severely ill patients were treated with methylprednisolone. There was no significant difference in average daily dose of methylprednisolone between the groups. Surprisingly, methylprednisolone was used for shorter durations in patients who died than in survivors. In addition, non-survivors had higher APACHE II scores. We also conducted multivariate CPHM analysis of critically ill patients. Hypertension, increased D-dimer levels, higher N/L, and high APACHE II score all increased the risk of death (Table 3), especially hypertension (HR 2.469; 95% CI [1.284–4.749]; *P* = 0.007; Fig. 2). Methylprednisolone dose and days of use had no significant effect on outcomes of COVID-19 patients.

**Table 3:** Cox proportional-hazard model analysis results for critically ill patients

	HR	95% CI	*P*-value
Hypertension	1.992	(1.019–3.895)	0.044
D-dimer	1.020	(1.010–1.031)	< 0.001
N/L	1.062	(1.017–1.108)	0.006
APACHE II score	1.117	(1.030–1.212)	0.007

Abbreviations: *HR* Hazard ratio; *CI* Confidence interval; *N/L* Ratio of neutrophils to lymphocytes; *APACHE II* Acute Physiology And Chronic Health Evaluation II. The following variables were incorporated into Cox proportional-hazard model analysis: sex, age, duration of methylprednisolone use (days), hypertension, N/L, D-dimer, and APACHE II score

### Results of PSM analysis

**Fig. 3:**
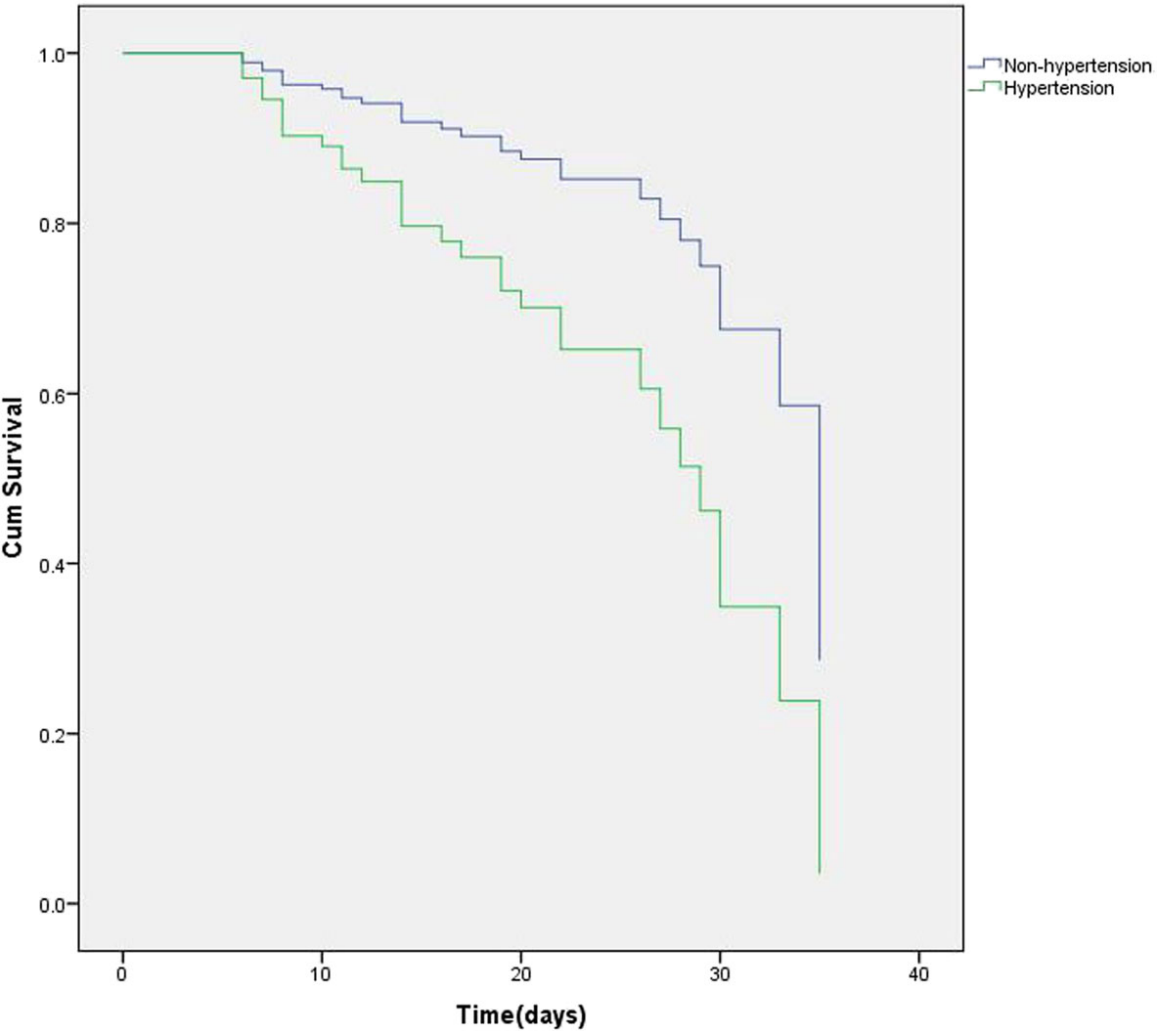
Cox regression survival plot according to hypertension after propensity score matching (PSM). Hypertension: hazard ratio (HR), 2.679; 95% confidence interval (CI), 1.237–5.805; *P* = 0.012

The abovementioned results indicated that hypertension had a great influence on the outcomes of patients with COVID-19. To decrease the effects of other confounding factors on the relationship between hypertension and outcomes, we used one-to-one PSM analysis. Before PSM, patients with hypertension differed significantly from those without on most of the covariates (Table 4). After PSM, between-group differences for all covariates were eliminated. In the matched group, we used CPHM analysis to estimate the effect of hypertension on patient outcomes. The results showed that patients with hypertension had significantly higher risk of death (HR 2.679; 95% CI [1.237–5.805]; *P* = 0.012; Fig. 3). In addition, elevated D-dimer levels (HR 1.025; 95% CI [1.011–1.039]; *P* < 0.001) and higher N/L (HR 1.107; 95% CI [1.053–1.164]; *P* < 0.001) could also increase patients’ mortality risk.

**Table 4:** Baseline characteristics of patients with and without hypertension

	Before PSM			After PSM		
	Non-hypertension (*n* = 142)	Hypertension (*n* = 84)	*P*-value	Non-hypertension (*n* = 54)	Hypertension (*n* = 54)	*P*-value
Males (%)	62 (42.66%)	51 (60.71%)	0.019	31 (57.71%)	30 (55.56%)	1.000
Age (years)	46.73 (15.20)	65.99 (12.81)	< 0.001	60.61 (11.64)	62.93 (13.74)	0.347
Onset time (day)	7.08 (4.00–10.00)	6.92 (4.00–9.75)	0.751	7.63 (4.27)	7.15 (4.25)	0.558
T2DM (%)	11 (7.75%)	34 (40.48%)	< 0.001	9 (16.67%)	15 (27.78%)	0.247
CHD (%)	5 (3.52%)	8 (9.52%)	0.078	5 (9.26%)	5 (9.26%)	1.000
COPD (%)	2 (1.41%)	4 (4.76%)	0.198	2 (3.70%)	2 (3.70%)	1.000
WBC (10^9^)	4.89 (3.63–5.90)	5.90 (4.21–7.27)	0.013	5.29 (4.01–7.15)	4.90 (3.66–6.16)	0.285
N/L	2.83 (1.61–5.07)	4.88 (2.95–7.78)	< 0.001	5.22 (3.27–7.72)	3.96 (2.17–6.49)	0.05
ALT (U/L)	19.80 (13.60–33.43)	20.75 (14.20–31.80)	0.766	25.65 (16.13–36.60)	24.65 (16.00–36.90)	0.649
AST (U/L)	21.00 (16.88–32.85)	26.85 (20.78–40.68)	0.008	32.15 (19.13–51.13)	25.90 (20.63–39.70)	0.197
ALB (g/L)	39.60 (5.27)	36.44 (4.70)	< 0.001	36.63 (4.96)	37.54 (4.44)	0.319
sCr (μmol/L)	64.90 (52.08–74.43)	74.35 (57.63–99.40)	0.001	71.55 (58.93–82.18)	72.05 (57.15–92.40)	0.792
BUN (mmol/L)	3.93 (3.07–5.02)	5.58 (3.66–8.09)	< 0.001	4.97 (3.75–6.43)	4.90 (3.62–7.14)	0.578
CRP (mg/dL)	1.16 (0.16–4.55)	6.10 (2.35–8.42)	< 0.001	4.74 (2.53–6.74)	4.13 (1.52–7.16)	0.541
D-dimer (μg/mL)	0.52 (0.25–1.54)	1.44 (0.62–6.81)	< 0.001	1.21 (0.50–8.59)	1.31 (0.61–6.57)	0.719

Abbreviations: *PSM* Propensity score–matching analysis; Onset time, time from onset to admission (days); *T2DM* Type 2 diabetes mellitus; *CHD* Coronary heart disease; *COPD* Chronic obstructive pulmonary disease; *WBC* White blood cell count; *N/L* Ratio of neutrophils to lymphocytes; *ALT* Alanine transaminase; *AST* Aspartate aminotransferase; *ALB* Serum albumin; *sCr* Serum creatinine; *BUN* Blood urea nitrogen; *CRP* C-reactive protein

**Fig. 4:**
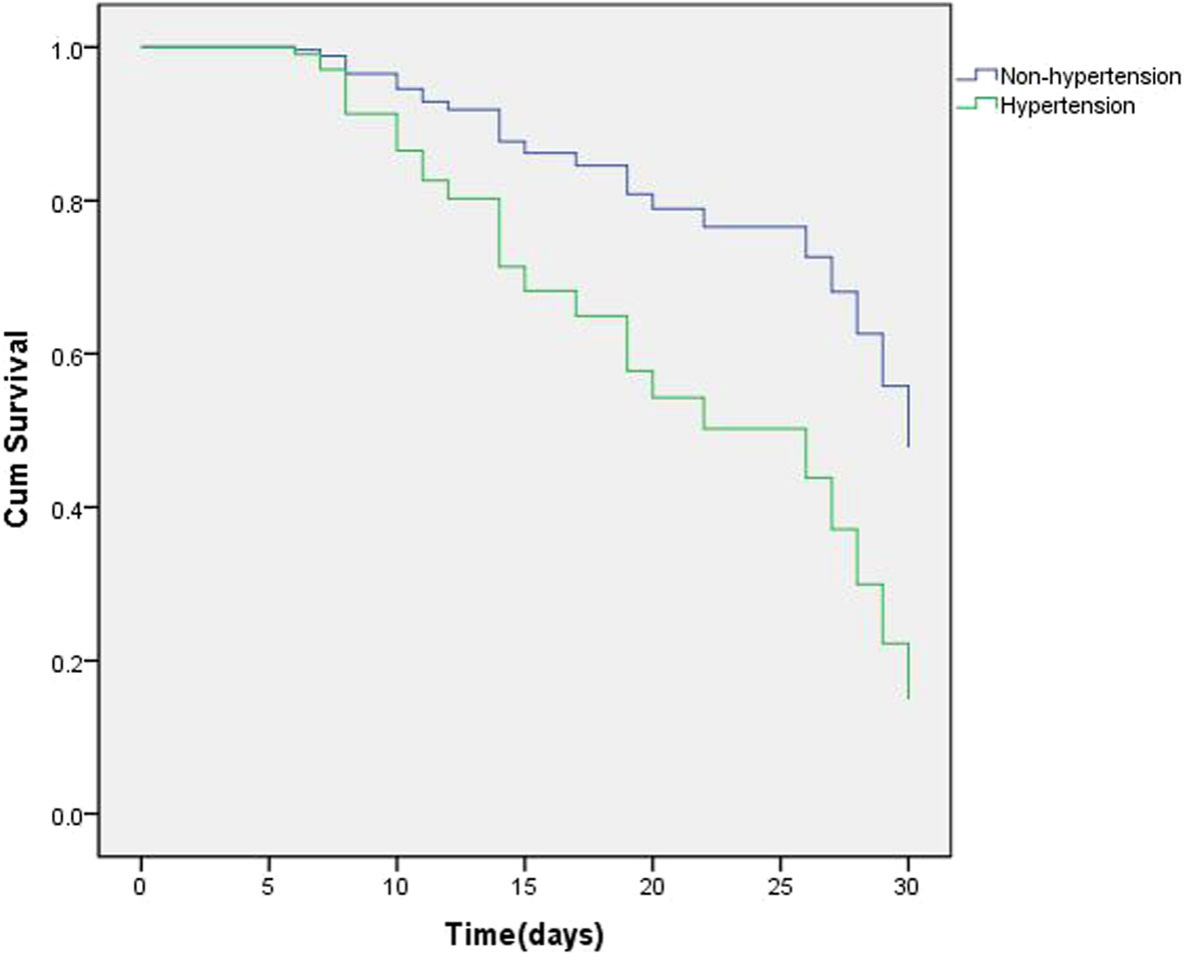
Cox regression survival plot according to hypertension after propensity score matching (PSM) in critically ill patients

We applied the same analysis to critically ill patients. Using PSM, we matched 31 patients with hypertension with 31 without. The results of prematching and postmatching were presented in the Table S3 in the Supplementary Appendix. After CPHM analysis, the results were similar to those we had obtained before matching (Table 5); the effect of hypertension on outcomes was still significant in critically ill patients (Fig.4).

**Table 5:** Cox proportional-hazard model analysis results after PSM for critically ill patients

	HR	95% CI	*P*-value
Hypertension	2.578	(1.142–5.820)	0.023
D-dimer	1.026	(1.012–1.039)	< 0.001
N/L	1.093	(1.036–1.153)	0.001
APACHE II score	1.185	(1.063–1.322)	0.002

Abbreviations: *PSM* Propensity score–matching analysis; *HR* Hazard ratio; *CI* Confidence interval; *N/L* Ratio of neutrophils to lymphocytes; *APACHE II* Acute Physiology And Chronic Health Evaluation II. The following variables were incorporated into Cox proportional-hazard model analysis: sex, age, duration of methylprednisolone use (days), average daily dose of methylprednisolone, hypertension, N/L, D-dimer, and APACHE II score

## Discussion

This retrospective cohort study showed that hypertension, increased levels of D-dimer, and higher N/L were associated with increased mortality in patients with COVID-19; high APACHE II score was also correlated with death in critically ill patients. Hypertension was the most prominent of the abovementioned risk factors in both cohorts. This study is the first to report risk factors for mortality in COVID-19 patients using PSM analysis. The results of PSM were the same as those we had already obtained before PSM analysis. Via PSM analysis, we provided robust evidence to support the association between hypertension and increased mortality.

In a study involving 191 patients with COVID-19, logistic-regression analysis showed that odds of in-hospital death were higher in patients with hypertension [4] . In another study conducted in January 2020, 11 of 99 patients with COVID-19 died, and 3 of the 11 had hypertension [7] . Yang et al. performed a systematic review and meta-analysis including 46,248 COVID-19 patients [5] . The results showed that the pooled odds ratio (OR) of hypertension in severe patients was 2.36 (95% CI [1.46–3.83]) compared with non-severe patients, indicating that hypertension might be a risk factor for severely ill patients. In addition, previous studies have shown that hypertension was also a factor in the prognoses of patients infected with other viruses. Hypertension was one predictor of ARDS in patients with severe acute respiratory syndrome, per the results of a study on clinical features and outcomes of severe acute respiratory syndrome [8] . Based on the six indices of Multilobular infiltration, hypo-Lymphocytosis, Bacterial coinfection, Smoking history, hyper-Tension, and Age, Guo et al. created the MuLBSTA score system based on a multivariate logistic-regression model to predict mortality in patients with viral pneumonia within 90 days [9] . The OR of hypertension is 2.39, and its MuLBSTA score is 2. A study on influenza suggested that patients with hypertension suffered a significantly higher risk of death (OR 1.49; 95% CI [1.10–2.10]) than patients who had no comorbidities [10] . Researchers reported the deaths of three people infected with avian-influenza H7N9 virus in Shanghai in 2013; two of them had hypertension [11] .

The role of hypertension in COVID-19 progression remains unclear. Our results showed that L count in the hypertensive group was significantly lower than in the non-hypertensive group, and the same was true in critically ill patients. We speculated that the poor outcome of the hypertension group might be associated with impairment of immune function caused by the decrease in lymphocytes. Consistent with our hypothesis, a recent study involving 61 patients with hypertension showed a significant decrease in peripheral blood CD3, CD4 and CD8 T-cell in hypertensive patients versus healthy controls [12] . Additionally, it has been found that some angiotensin-converting enzyme inhibitor and angiotensin II receptor blocker drugs could significantly increase the expression of angiotensin-converting enzyme 2 (ACE2) messenger ribonucleic acid in rat hearts [13] . As ACE2 is the receptor of SARS-CoV-2 in humans, the increase in ACE2 messenger ribonucleic acid expression could aggravate the illness. Further study is needed to determine whether certain antihypertensive drugs contribute to poor clinical outcomes in patients with COVID-19. Finally, hypertension and other chronic diseases share some common characteristics, such as a proinflammatory state and attenuation of the innate immune response. Therefore, the body cannot effectively control the virus in the early stage, which leads to aggravation of illness in COVID-19 patients [5, 14] . Further study is needed on the role of hypertension in this disease.

In addition to hypertension, we found that high D-dimer levels and high N/L were associated with increased in-hospital mortality of COVID-19 patients in general and of critically ill ones in particular, which was consistent with previous studies. N/L might reflect levels of both inflammation and immune response of the human body during viral infection. Some studies have shown that coronaviruses consume many immune cells, leading to a great reduction in lymphocytes, which inhibits cellular immune function. Damage to T cells might be an important factor leading to deterioration of patients [7, 15] . Neutropenia represents the aggravation of inflammatory response and cytokine storm, while lymphocytes are the main immune-active cells in the body, and L count is an early marker of physiological stress and systemic inflammation [3, 16, 17] . Wang et al. have reported that in 33 patients with COVID-19, survivors had higher N counts and lower L counts than non-survivors [16] . Neutrophils are proven to be related to the development of ARDS and progression from ARDS to death in patients with COVID-2019^3^. In a study by Zhou et al., univariate analysis showed that decreased L count was associated with in-hospital death in such patients [1] .

Consistent with our results, a recent study of 191 patients with COVID-19 showed that D-dimer > 1 μg/ml was associated with fatal outcomes [1] . In the other two studies including 33 and 183 patients with COVID-19, D-dimer levels were significantly higher in non-survivors versus survivors [16, 18] . Recently, a bivariate Cox regression analysis also suggested that higher D-dimer levels were related to the development of ARDS and progression from ARDS to death in patients with COVID-19 (HR 1.03; 95% CI [1.01–1.04] and HR 1.02; 95% CI [1.01–1.04], respectively) [3] . The mechanism of fatal outcome in patients with higher D-dimer could be that systemic pro-inflammatory cytokine responses induce the production of procoagulant factors and hemodynamic changes [1, 19] .

In addition, our study showed that a high score on the APACHE II, a tool for estimating disease severity and mortality in-hospital in critically ill patients, was associated with increased mortality in patients with severe COVID-19. Patients with high APACHE II scores have also been found to have higher risk of death in other studies. These studies suggest that clinicians should pay more attention to patients with high APACHE II scores.

The following shortcomings existed in our research. First, this was a single-center study with a limited number of cases included. At this writing, the epidemic has spread throughout the world. A multi-center study should be carried out that includes other hospitals in Wuhan, other cities in China, and other countries worldwide, as it will yield a more comprehensive understanding of the factors affecting the outcomes of COVID-19 patients. Second, as this study was retrospective, the indicators included in our study were finite. The findings of chest CT, viral load in the respiratory tract or circulation, angiotensin II, and lymphocyte subsets could be potential indicators of outcomes of patients, which should be further confirmed by more studies. Finally, due to failure to record the classification, stratification, and use of antihypertensive drugs in patients with hypertension, it is necessary to further explore whether these factors could influence patient outcomes.

## Conclusion

Hypertension, high D-dimer levels, and a high neutrophil-to-lymphocyte ratio all increased mortality in patients with COVID-19, with hypertension being the most prominent factors. Clinicians should note these findings. Multicenter, prospective studies with large samples are needed to further validate the findings.

## Supplementary information

**Additional file 1:Table S1.** shown thant results of univariate Kaplan-Meier analysis for all participators. **Table S2.** listed the baseline characteristics of critically ill patients. Baseline characteristics of critically ill patients with and without hypertension were presented in the **Table S3.**

## Data Availability

The datasets analysed during the current study are not publicly available due to hospital patient data protection policy but are available from the corresponding author on reasonable request.

## Abbreviations

ACE2: Angiotensin-converting enzyme 2
ALB: Albumin
ALT: Alanine transaminase
APACHE II: Acute physiology and chronic health evaluation ii
ARDS: Acute respiratory distress syndrome
AST: Aspartate aminotransferase
BUN: Blood urea nitrogen
CHD: Coronary heart disease
CI: Confidence interval
COPD: Chronic obstructive pulmonary disease
COVID-19: Coronavirus disease 2019
CPHM: Cox proportional-hazard model
CRP: C-reactive protein
CT: Computed-tomography
HR: Hazard ratio
IQRs: Interquartile ranges
K–M: Kaplan–Meier
L: Lymphocytes
N: Neutrophils
N/L: Ratio of neutrophils to lymphocytes
OR: Odds ratio
PSM: Propensity score–matching
SARS-CoV-2: Severe acute respiratory syndrome coronavirus 2
sCr: Serum creatinine
SD: Standard deviation
SpO2: Oxygen saturation
T2DM: Type 2 diabetes mellitus
WBCs: White blood cells
WHO: World health organization
