# Supplementary material for: Effect of hypertension on outcomes of adult inpatients with COVID-19 in Wuhan, China: a propensity score–matching analysis
Source: Respir Res. 2020 Jul 6;21:172. doi: 10.1186/s12931-020-01435-8 (PMC7336415; doi:10.1186/s12931-020-01435-8)
Supplement: Supplementary file 1 — Additional file 1:Table S1. shown thant results of univariate Kaplan-Meier analysis for all participators. Table S2. listed the baseline characteristics of critically ill patients. Baseline characteristics of critically ill patients with and without hypertension were presented in the Table S3. [file 12931_2020_1435_MOESM1_ESM.docx]

**Table S1: Results of univariate Kaplan-Meier analysis**

|  | Results classified by median | *P*-value |
| --- | --- | --- |
| N/L | <3.6464 | 0.002 |
|  | ≥3.6464 |  |
| CRP (mg/dL) | <2.555 | <0.001 |
|  | ≥2.555 |  |
| AST (U/L) | <23.000 | 0.001 |
|  | ≥23.000 |  |
| ALB (g/L) | <38.600 | 0.002 |
|  | ≥38.600 |  |
| sCr (μmol/L) | <66.300 | 0.120 |
|  | ≥66.300 |  |
| BUN (mmol/L) | <4.1900 | <0.001 |
|  | ≥4.1900 |  |
| D-dimer (μg/mL) | <0.8300 | <0.001 |
|  | ≥0.8300 |  |
| Age (years) | <56.000 | <0.001 |
|  | ≥56.000 |  |
| T2DM | N/A | 0.012 |
| Hypertension | N/A | <0.001 |
| CHD | N/A | 0.255 |

Abbreviations: N/L, ratio of neutrophils to lymphocytes; CRP, C-reactive protein; AST, aspartate aminotransferase; ALB, serum albumin; sCr, serum creatinine; BUN, blood urea nitrogen; T2DM, type 2 diabetes mellitus; CHD, coronary heart disease.

**Table S2: Baseline characteristics of critically ill patients**

|  | Survivors (n = 55) | Non-survivors (n = 50) | *P*-value |
| --- | --- | --- | --- |
| Males (%) | 34 (61.82%) | 31 (62%) | 1.000 |
| Age (years) | 60.82 (11.66) | 67.82 (15.73) | 0.011 |
| Onset time (days) | 7.11 (3.91) | 6.54 (3.71) | 0.447 |
| Hypertension (%) | 25 (45.45%) | 37 (74%) | 0.005 |
| T2DM (%) | 16 (29.09%) | 17 (34%) | 0.675 |
| CHD (%) | 5 (9.09%) | 7 (14%) | 0.544 |
| COPD (%) | 2 (3.64%) | 3 (6%) | 0.667 |
| WBC (10^9^) | 5.66 (3.92–7.04) | 5.73 (4.18–7.95) | 0.388 |
| N/L | 6.31 (3.12) | 9.24 (7.84) | 0.016 |
| ALT (U/L) | 25.80 (15.20–38.70) | 21.05 (14.33–29.48) | 0.096 |
| AST (U/L) | 29.40 (25.00–50.60) | 31.50 (22.40–44.33) | 0.646 |
| ALB (g/L) | 36.80 (5.18) | 35.55 (4.30) | 0.183 |
| sCr (μmol/L) | 72.30 (59.30–85.30) | 76.00 (58.00–109.20) | 0.233 |
| BUN (mmol/L) | 4.94 (3.50–6.60) | 6.60 (4.60–10.58) | <0.001 |
| CRP (mg/dL) | 6.10 (2.78–8.20) | 6.10 (3.53–8.84) | 0.669 |
| D-dimer (μg/mL) | 2.65 (0.66–8.48) | 6.21 (1.27–19.73) | 0.007 |
| corticosteroid use (d) | 10.60 (7.14) | 7.98 (5.43) | 0.036 |
| Mean corticosteroid (mg/d) | 44.57 (30.86) | 49.34 (31.80) | 0.437 |
| APACHE II score | 8.51 (3.32) | 12.44 (3.41) | <0.001 |

Abbreviations: Onset time, time from onset to admission (days); T2DM, type 2 diabetes mellitus; CHD, coronary heart disease; COPD, [chronic](D:/Program%20Files%20(x86)/Youdao/Dict/8.7.0.0/resultui/html/index.html#/javascript:;) [obstructive](D:/Program%20Files%20(x86)/Youdao/Dict/8.7.0.0/resultui/html/index.html#/javascript:;) [pulmonary](D:/Program%20Files%20(x86)/Youdao/Dict/8.7.0.0/resultui/html/index.html#/javascript:;) [disease](D:/Program%20Files%20(x86)/Youdao/Dict/8.7.0.0/resultui/html/index.html#/javascript:;); WBC, white blood cell count; N/L, ratio of neutrophils to lymphocytes; ALT, alanine transaminase; AST, aspartate aminotransferase; ALB, serum albumin; sCr, serum creatinine; BUN, blood urea nitrogen; CRP, C-reactive protein; corticosteroid use (d), duration of methylprednisolone use (days); Mean corticosteroid, average daily dose of methylprednisolone; APACHE II, Acute Physiology And Chronic Health Evaluation II.

**Table S3: Baseline characteristics of critically ill patients with and without hypertension**

|  | Before PSM | | | After PSM | | |
| --- | --- | --- | --- | --- | --- | --- |
|  | Non-hypertension  (n = 43) | Hypertension (n = 62) | *P*-value | Non-hypertension (n = 31) | Hypertension (n = 31) | *P*-value |
| Males (%) | 27 (62.79%) | 38 (61.29%) | 1.000 | 18 (58.06%) | 20 (64.52%) | 0.795 |
| Age (years) | 58.53 (13.51) | 68.05 (13.30) | 0.001 | 60.00 (12.98) | 62.74 (14.49) | 0.436 |
| Onset time (day) | 7.09 (4.01) | 6.66 (3.68) | 0.570 | 7.26 (4.33) | 6.29 (3.80) | 0.353 |
| T2DM (%) | 7 (16.28%) | 26 (41.94%) | 0.006 | 7 (22.58%) | 10 (32.26%) | 0.570 |
| CHD (%) | 4 (9.30%) | 8 (12.90%) | 0.757 | 3 (9.68%) | 2 (6.45%) | 1.000 |
| COPD (%) | 1 (2.33%) | 4 (6.45%) | 0.646 | 0.00 | 2.00 | N/A |
| WBC (10^9^) | 5.26 (3.64–6.70) | 6.06 (4.30–7.43) | 0.116 | 5.66 (3.67–7.51) | 6.15 (4.31–7.49) | 0.583 |
| N/L | 5.97 (4.44–8.75) | 6.18 (3.70–10.05) | 0.820 | 5.81 (4.13–8.61) | 7.16 (4.51–12.71) | 0.418 |
| ALT (U/L) | 27.30 (16.70–38.70) | 19.40 (14.05–29.48) | 0.013 | 25.20 (15.50–34.20) | 23.70 (14.20–36.90) | 0.414 |
| AST (U/L) | 33.00 (27.10–58.60) | 28.50 (22.00–42.15) | 0.017 | 32.80 (27.10–45.60) | 29.40 (21.00–44.70) | 0.208 |
| ALB (g/L) | 36.60 (5.39) | 35.93 (4.36) | 0.481 | 35.74 (4.65) | 36.45 (4.73) | 0.554 |
| sCr (μmol/L) | 71.60 (59.90–77.60) | 77.10 (57.53–111.50) | 0.144 | 68.50 (57.90–76.00) | 80.90 (58.00–114.30) | 0.108 |
| BUN (mmol/L) | 5.20 (3.80–6.60) | 5.85 (4.12–9.04) | 0.077 | 5.49 (3.80–6.40) | 5.80 (4.14–8.46) | 0.360 |
| CRP (mg/dL) | 6.10 (3.56–7.55) | 6.10 (3.15–9.01) | 0.338 | 6.10 (3.56–7.56) | 6.10 (3.29–8.49) | 0.612 |
| D-dimer (μg/mL) | 3.38 (0.83–12.16) | 3.31 (1.14–9.71) | 0.767 | 2.21 (0.83–9.06) | 4.28 (1.54–11.93) | 0.151 |
| corticosteroid use (d) | 11.00 (9.00–17.00) | 7.00 (2.00–12.00) | 0.002 | 10.00 (6.00–14.00) | 10.00 (7.00–12.00) | 0.667 |
| Mean corticosteroid (mg/day) | 48.89 (40.00–66.67) | 40.00 (33.31–60.71) | 0.042 | 47.27 (40.00–67.27) | 40.00 (38.33–57.14) | 0.173 |
| APACHE II score | 8 (6–11) | 11 (9–14) | <0.001 | 8 (6–12) | 10 (8–13) | 0.086 |

Abbreviations: Onset time, time from onset to admission (days); T2DM, type 2 diabetes mellitus; CHD, coronary heart disease; COPD, [chronic](D:/Program%20Files%20(x86)/Youdao/Dict/8.7.0.0/resultui/html/index.html#/javascript:;) [obstructive](D:/Program%20Files%20(x86)/Youdao/Dict/8.7.0.0/resultui/html/index.html#/javascript:;) [pulmonary](D:/Program%20Files%20(x86)/Youdao/Dict/8.7.0.0/resultui/html/index.html#/javascript:;) [disease](D:/Program%20Files%20(x86)/Youdao/Dict/8.7.0.0/resultui/html/index.html#/javascript:;); WBC, white blood cell count; N/L, ratio of neutrophils to lymphocytes; ALT, alanine transaminase; AST, aspartate aminotransferase; ALB, serum albumin; sCr, serum creatinine; BUN, blood urea nitrogen; CRP, C-reactive protein; corticosteroid use (d), duration of methylprednisolone use (days); Mean corticosteroid, average daily dose of methylprednisolone; APACHE II, Acute Physiology And Chronic Health Evaluation II.
